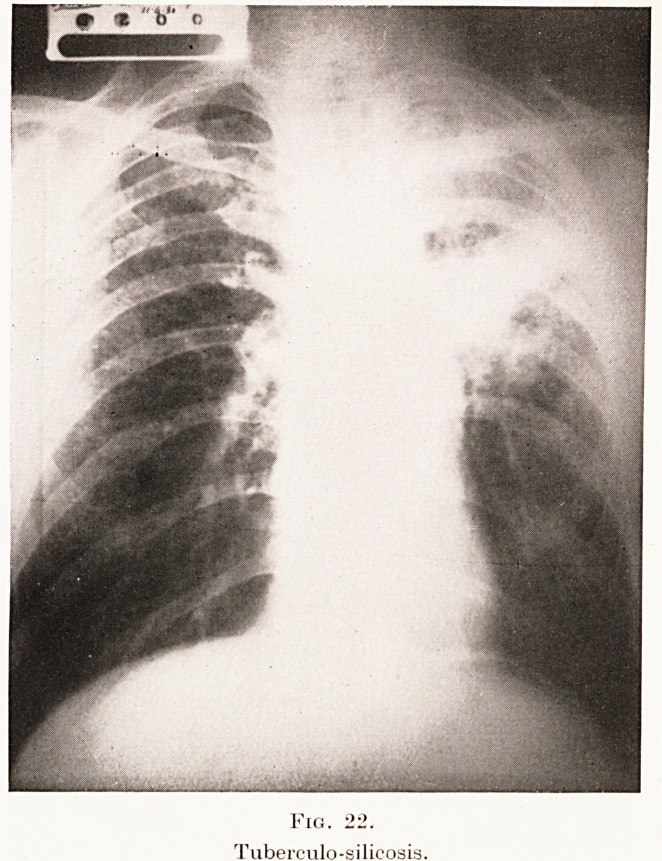# Silicosis: II.—Radiology and Pathology

**Published:** 1931

**Authors:** G. B. Bush

**Affiliations:** Assistant Radiologist, Bristol General Hospital


### SILICOSIS.

### II.—RADIOLOGY AND PATHOLOGY.

BY

G. B. Bush, M.B., Ch.B., D.M.R.E.,
*Assistant Radiologist, Bristol General Hospital.*

---

I hope to be able to show you that a study of silicosis from two angles, the pathological and the radiological, is of more than academic interest, for in radiology we have a method, by means of serial X-ray examinations at different stages of the disease, of watching the pathological processes at work during life, and of correlating the findings with clinical symptoms and the degree of disability.

In a case suspected, on clinical grounds, of silicosis, the two deciding points are (1) a history of exposure to silica dust, and (2) X-ray findings. Either alone is not enough. If the disease is complicated by infection, such as tubercle (as it often is) the matter becomes more difficult ; but in uncomplicated silicosis in the second or third stages, as I shall point out, the X-ray appearances are fairly characteristic.

Before we consider what actually occurs in the lung a brief survey of the *lymphatic system of the lungs and pleura* is at first necessary, since it is this system which is primarily affected in this disease. So far as we know anything about them, the following are the main points. There are no lymphatics in the alveoli themselves. Small nodules of lymphoid tissue are found in intimate relation with the distal ends of the terminal bronchioles. There are two main groups of lymphatic vessels, deep and superficial. The deep lymphatics start at the terminations of the smallest bronchioles, and run in complex interlacing channels along with the bronchi and blood-vessels to the tracheo-bronchial and paratracheal glands at the roots of the lungs. The superficial lymphatics, which are said to have no anastomosis with the deep vessels, start on the periphery of the lung, and course along as a fine network between the lung and the visceral pleura, ramifying in the interlobar fissures, and eventually finding their way to another set of glands, situated at the hilum, where some anastomosis with the deep system takes place.

There is also a fine network of lymphatic vessels more intimately connected with the larger bronchi, consisting of two networks—a submucous, beneath the mucous membrane, and the peribronchial outside the walls of the bronchi. The importance of the distribution of the lymphatics will be evident later.

The course of affairs in the *development of silicosis* is as follows: dust enters the lungs by the air passages, and may set up a transitory and mild degree of bronchitis. It has been shown repeatedly that it is only the smallest particles of siliceous dust, having a diameter of one to three microns (one to three twenty-five thousandths of an inch), which gain entrance to the alveoli of the lung and produce the disease. Those which do not reach the alveoli are probably caught by the mucous secretions of the air-tubes, and carried away by the ciliated epithelium and by expectoration. In the early stages of the disease, at any rate, they do not appear to cause any marked degree of bronchitis, nor any recognizable peribronchial fibrosis. The finest dust-particles are taken up by certain phagocytes derived from the alveolar epithelium, and carried by them to the nearest lymphoid tissue, and hence into the lymphatic vessels, *i.e.* they are deposited first in and around the lymph-nodules, which lie, as we have seen, in close relation with the terminal bronchioles, and also immediately under the visceral pleura. (Plate VI, Fig. 1.)

Gye and Kettle consider that finely-divided silica, which probably gets converted into a colloidal form in the lungs, acts as a slow cell-poison, sufficiently slow in its action to promote a progressive local fibrosis, but in the absence of infection not sufficient to produce necrosis. (Plate VI, Figs. 2, 3, 4.)

Heffernan believes that silicosis is the result of a local physico-chemical action of hydrated silica on pulmonary tissue, the silica absorbing moisture from neighbouring cells and, in effect, "drying them up." The dust-cells are also probably disintegrated by the same process, the peculiar action of silica dust in the form of particles of from 0·5 to 5 microns depending on its solubility. The speed of development depends on the rapidity of formation of fresh silica hydrosol. Alkalies accelerate the formation (*e.g.* in silica scouring-powders), while carbon and clay retard it.

From the lymph-nodes some of the laden dust-cells are next carried along a further set of lymph-channels, and eventually many of them reach the tracheo-bronchial and paratracheal glands at the roots of the lungs, where further fibrotic changes tend to take place. As the dust-cells congregate, through repeated exposure of the patient, the lymph vessels tend to dilate and a certain amount of general lymph-stasis occurs. This stasis is probably only a transitory phenomenon, but one often sees evidence of it in radiographs of early stages of the disease, showing as an increase in the number and extent of the linear "striations" radiating from the hilar areas, so that practically the whole of both lung-fields is occupied by what is sometimes referred to as a "fine general arborization." In a radiograph of a normal lung the linear striations should only be seen extending about two-thirds of the way across the lung-fields, the peripheral third appearing clear and free from any but very faint linear shadows only seen on a good skiagram.

Radiologically and pathologically it is convenient to divide the disease into three stages: early, intermediate and advanced uncomplicated silicosis; but this classification will probably have to be modified in the light of recent studies. It is found that the three stages to be described, while holding in many cases, do not always follow each other, the type of fibrosis and its progress depending on the type of dust-industry, time-factors and the intensity of the dust inhaled in a given period. As new occupational fibroses are studied classification has become more difficult. It will be convenient, however, to describe three stages which are frequently found, and to discuss their modifications later.

In the *first stage* the hilum shadows are accentuated, denser and more extensive than normal. The pulmonary striations are also accentuated and extend well out to the periphery of the lung. This appearance generally begins in the right lower zone, and later extends symmetrically over both lung-fields. (Plate VIII, Fig. 7.) This picture is probably due, in this disease, to the clogging of the lymphatics with laden dust-cells, and to a degree of dilatation of these vessels with some slowing up of the lymph-stream. There is no alteration in diaphragmatic excursion. The appearance at this stage is not characteristic; a similar picture may be produced by acute or chronic bronchitis (Plate VIII, Fig. 8), passive congestion of the lungs from cardiac decompensation (Plate IX, Fig. 9), irritation from certain gases (Plate IX, Fig. 10), and more rarely by metastatic or primary mediastinal malignancy.

The *second stage* is characterized by mottling throughout the lungs, due to small fibrotic nodules varying in size from a pinhead to a pea. The shadows are not very dense, are rather fluffy in outline, and have been likened to a snowstorm. This process usually starts in the right lung near the hilum, but soon assumes a more or less generalized distribution. There may be slight restriction of diaphragmatic movement. During this stage the accentuation of the hilum shadows and of the linear lung-striations usually disappears.

Describing this transition from linear markings to nodular fibrotic infiltrations, Steuart says: "There is an apparent segmentation of linear markings which now present themselves as numerous small, discrete, but clearly-aggregated miliary tubercles. This change from reticulation to nodulation usually progresses uniformly throughout the entire lung, and, as the condition advances, the nodules grow in size but apparently decrease in number."

Radiographically, then, the picture at this stage is very similar to miliary tuberculosis, except that the miliary infiltrations are due to fibrotic nodules and not to the tubercles of tuberculosis. The transition of the picture from linear markings to nodule-formation is explained by the clogging of the lymph-channels with dust-cells, stagnation of the stream, and the supervention of fibrosis. The radiographic appearances of the typical "second stage" of silicosis are shown in Plate X, Figs. 11, 12, and the macroscopic features in Plate VII, Fig. 5.

The condition has to be differentiated from miliary tuberculosis, acute (Plate XI, Fig. 13) or chronic (Plate XI, Fig. 14), broncho-pneumonia, carcinomatosis (Plate XII, Fig. 15), and some rare mycotic infections (sporotrichosis). A careful consideration of the history and clinical findings is of the utmost importance in coming to a conclusion.

In the *third stage* the fibrosis extends, and the isolated nodules enlarge and coalesce into coarser nodules and patches of fibrosis. There is probably a leakage of dust-cells through the walls of the distended and blocked lymphatics, causing a more diffuse fibrosis in the interstitial lung tissues. This is possibly the cause of a re-appearance in this stage of the accentuated linear markings and dense hilum shadows seen in the radiograph. It is suggested by some observers that, with the blocking of the lymphatics of the lung, the lymph-flow is dammed back, and tends to find its way to the hilum by the pleural route. This gives rise in extreme cases to thickening of the pleura, adhesions and occasionally pleural effusion. Pleural involvement usually results in restriction of the excursion of the diaphragm. In rapidly-progressing cases this interstitial and pleural involvement may occur early, and the nodular stage be masked or fail to develop. This has been observed in the case of granite-cutters.

The skiagrams in Plates XII, XIII, Figs. 16, 17, from cases in the "third stage," illustrate these points. In the radiographic differential diagnosis these appearances have to be distinguished from those found in chronic fibroid phthisis, fibrosis from chronic infections (Plate XIII, Fig. 18), chronic interstitial pneumonia of

PLATE VI.

Fig. 1.

Photomicrograph. Pigmented cell aggregates.

A longitudinal section of a terminal bronchiole and its vestibule is shown. Note the accumulation within the walls of these structures of very numerous "dust cells," amidst which a few fibroblasts appear.

Fig. 2.

Photomicrograph. Commencing nodule formation.

A terminal bronchiole is shown with a related aggregate of pigmented "dust cells" in which a commencing nodular silicotic fibrosis is seen. The focus of fibrosis is still in the cellular stage.

Fig. 3.

Photomicrograph. Fully formed single small "non-infective" silicotic nodule.

Note that the simple silicotic islet consists of a central area, composed of well-formed dense fibrous tissue with scanty cells and little pigment; most of the pigment is lying free. At the periphery of the islet the arrangement is that of concentric laminæ and the tissue is fibro-cellular. The islet is surrounded by an accumulation of pigmented "dust cells."

FIG. 4.

Photomicrograph. Composite "non-infective" silicotic nodule.

The section shows several small silicotic islets which were originally separate. The close proximity of their sites of origin has brought them into contact to form a "composite" structure. Note the sharp separation of the nodule from the surrounding lung tissue, which shows no significant change.

FIG. 5.

Macroscopic appearances in a moderate degree of silicosis of simple type.

Numerous discrete pigmented islets appear on the cut surface of the lung. They are mostly "small" and a considerable proportion are the seat of a palpable nodular fibrosis. The root glands are pigmented and fibrosed. A portion of pleura, with a few sub-pleural plaques, is visible on the right.

FIG. 6.

Macroscopic appearances in an advanced degree of silicosis of infective type. (Tuberculo-silicosis.)

Note the large irregularly-shaped nodules and in the upper lobe two areas of massive fibrosis of infective type. Material from the root glands and from the massive areas in this case was inoculated into guinea-pigs, and the animals in each case died from generalized tuberculosis.

PLATE VIII.

Fig. 7.
Silicosis. Early stage.

Fig. 8.
Bronchitis and emphysema.

PLATE IX.

Fig. 9.
Passive congestion of lungs in heart disease.

Fig. 10.
Fibrosis following gas-inhalation.

PLATE X.

Fig. 11.
Silicosis. Commencing second stage.

Fig. 12.
Silicosis. Second (nodular) stage.

PLATE XI.

Fig. 13.

Acute miliary tuberculosis.

Fig. 14.

Chronic miliary tuberculosis.

PLATE XII.

FIG. 15.
Carcinomatosis with effusion.

FIG. 16.
Silicosis. Advanced (third) stage.

FIG. 17.
Silicosis. Advanced stage; marked pleurisy.

FIG. 18.
Fibrosis from Chronic Infection (non-Tuberculous).

PLATE XIV.

FIG. 19.
Multiple deposits of secondary sarcoma.

FIG. 20.
Silicosis. Late (third) stage. Infective focus in left middle zone.

PLATE XV.

Fig. 21.
Tuberculo-silicosis. Cavity at left apex.

Fig. 22.
Tuberculo-silicosis.

pyogenic origin, tuberculous broncho-pneumonia, and multiple secondary deposits of new growth. Plate XIV, Fig. 19, is from a case of sarcomatosis. The question of the symmetry of the lesions and a careful consideration of the history, etc., will generally decide the diagnosis.

In cases of simple, uncomplicated silicosis the fibrotic changes may take anything from two to thirty years to develop, depending on the percentage of silica in the dust inhaled and on its intensity in a given period. Some early cases in the first or commencing second stage were observed recently in America to show actual regression of the disease during several months' enforced idleness during a strike. In other words, there seems to be a stage, in the absence of infection, where the pathological changes may actually regress, and a cure with little or no fibrosis may result. But this has rarely happened in the past, although with increasing knowledge preventive measures and early routine examinations we may be able to catch cases early enough before irreparable damage has been done.

These latter observations on the regression of the radiographical features of the early stages of the disease tend to support the foregoing interpretation of the shadows seen, namely, that the increase of linear radiations and the earliest stages of nodule formation are due to lymphatic congestion with dust-cells rather than to actual fibrosis. If the inhalation has not been excessive in point either of time or of intensity, such dust-cells can be eventually got rid of by normal physiological processes. Fibrosis, however, once established, leaves permanent scars, and the degree of disability will depend on the amount of the "physiological reserve" of the lungs which has been left intact, assuming that no super-added infection has complicated the picture. That the intensity of silica dust inhaled in a given period is as important as the time-factor is illustrated by four cases which were recently reported. In spite of relatively short exposures, sufficient dust was deposited in the lungs to set up a progressive fibrosis, which only produced symptoms many years after; *e.g.* one developed symptoms twenty-three years after exposure of only four months; another developed symptoms ten years after exposure of two years.

The main *symptom* generally complained of is an increasing dyspnœa on exertion with occasionally some unproductive nocturnal cough.

The *physical signs* may be few and indefinite, being mainly those of a degree of emphysema, with restricted air-entry. These cases may succumb eventually to right heart failure, due to the emphysema (rather than the fibrosis) interfering with the pulmonary circulation.

The degree of disability may not be great until the third stage is reached, but if infective complications are super-added, even in the early stages of the disease, the story is a very different one. This brings me to the point where we must discuss the disease *when complicated by infection*, often referred to by the South African authorities by the term "infective silicosis."

The infection is nearly always tuberculous in nature, either due to the flaring up of an old focus already present in the lung, a focus which has lain dormant but is potentially active, or to the inhalation of fresh bacilli, which find a happy hunting-ground among the already partially-poisoned cells of a silicotic lung.

Subjects of advanced silicosis more often contract tuberculosis than pneumonia, but the latter is more often found as a complication in earlier cases, where the inhalations are copious and the fibrosis slight (Belt). Two new pathological processes now make their appearance, namely focal necrosis and a tendency to excessive fibrous tissue formation. It is probable that the old text-books were wrong, and that necrosis never takes place in silicotic nodules in the absence of super-added infection. With regard to the excessive fibrous tissue formation, it is generally found that tuberculosis and silicosis together produce greater fibrosis than either alone. The fact that the silicotic lung is peculiarly prone to invasion by the tubercle bacillus has long been known, but is none the less interesting as a pathological problem. There is a mechanical factor at work, namely that the lymphatic drainage system of the lungs is already considerably impaired. In addition, many of the cells are poisoned by the soluble silica, and thus the cellular defensive mechanism of the lung is largely thrown out of action. Further, it is possible that the "adsorptive" powers of colloidal silica referred to previously may bind the complement of human serum, and so antibodies are prevented from attacking the bacilli.

*Table of a Series of Cases Examined* (1929–31).

| | |
|---|---|
| Total number examined for possible silicosis .. .. .. .. | 124 |
| Total number found to have definite silicosis .. .. .. .. | 91 |

Of these 91 cases, positive for silicosis, 71·6 per cent. were cases uncomplicated by infection; 28·4 per cent. were infective cases, probably tuberculous.

Of the uncomplicated cases of silicosis, there were :—

| | |
|---|---|
| Stage 1 (early cases) .. .. .. | 11 |
| Stage 2 .. .. .. .. .. | 26 |
| Stage 3 .. .. .. .. .. | 28 |

Of the infective cases over 90 per cent. were in the advanced (third) stage.

Of the 33 remaining cases of the series which were negative for silicosis, 12 had chronic fibroid phthisis, and 21 had a slight degree of chronic bronchitis, emphysema or old pleurisy.

The interesting point brought out by this analysis is the fact that infective complications were commonest in those cases which were classified as belonging to the third or late stage of the disease. It is possible that the radiographic appearances hitherto described as typical of this late stage may be, after all, but modifications of the so-called second stage, due to infection, and that once the typical, discrete, nodular and linear shadows of uncomplicated silicosis begin to become blurred and confluent and massive shadows appear, then we must deduce that infection is already present, and that if followed up and re-examined at intervals these cases will soon show signs of increasing consolidation, caseation and necrosis, with cavity-formation, or a rapidly-progressing fibrosis such as is never seen in silicosis or tuberculosis alone.

In pre-war days dust conditions were very much worse than now, fibrosis was relatively more marked in silicotic lungs, and cases of advanced silicosis were often seen which showed no clinical evidence of infection. These patients died from heart-failure, with ascites, œdema and marked dyspnœa with cyanosis. Such cases are not so often seen nowadays, but cases in the late stage who have escaped active breakdown and are still classed as cases of simple silicosis nevertheless show radiographic and often clinical signs of a latent infection. Ultimately the infective element tends to gain the upper hand, and the condition becomes one of definite "tuberculosis with silicosis."

The radiographic recognition of "tuberculosis with silicosis" is sometimes very difficult, but serial studies and the observation of rapid changes will make the diagnosis more certain. The radiograph should show the characteristic signs of both diseases. In addition to the generalized and symmetrically distributed signs of silicosis in one or other of its stages, irregular blurring of the discrete nodular shadows is seen, together with extensive local opacities representing tuberculous consolidation, the latter often showing translucent areas of cavitation within them and showing an asymmetrical distribution. (Plates XIV, XV, Figs. 20, 21, 22 and Plate VII, Fig. 6.)

The clinical history and findings must be carefully considered in connection with the radiographic findings in these cases, since these appearances can be closely simulated by those found in a case of chronic fibroid phthisis with a recent bronchogenic spread. The latter may produce diffusely-scattered discrete lesions which mimic the coarse mottling of the later stages of silicosis, but they are usually less generalized and less well defined. First-class skiagrams are essential.

In a few cases the diagnosis must remain presumptive only, but here serial radiographs at suitable intervals are of great value in coming to a conclusion.

An interesting point, which needs elucidation, arises from a study of cases of silicosis, namely the fact that bronchiectasis is rarely found in association with the disease when uncomplicated by infection; and even in those cases where tuberculosis has supervened bronchial dilatation appears to be rare, when one considers how frequently it is found in other cases of lung-fibrosis following an acute or a chronic infection.

The explanation probably lies (1) in the peculiar character and distribution of the fibrosis in silicosis, and (2) in the fact that pathological changes are relatively slight in the walls of the air-tubes as a result of the dust-inhalation, whereas, as a sequel to infections, degenerative changes in the bronchi may be marked.

There is not time here for more than a few remarks about the allied condition of lung-fibrosis known as *asbestosis*. The various forms of commercial asbestos are essentially a mixture, in varying proportions, of hydrated silicates of magnesium, aluminium, and iron, the former preponderating. In its main clinical features asbestosis closely resembles silicosis, but it differs from it (1) in the mode of distribution of the fibrous tissue in the lungs, this appearing more in the basal areas and in the interstitial tissues of the lung; (2) in its rather more rapid development; (3) in its radiological features, the fibrosis beginning to show itself in the basal areas as an increase of the linear radiations, and a diffuse blurring of detail, rather than the nodular formation seen in silicosis: the pleura also shows earlier signs of involvement, diaphragmatic restriction of movement appears early, and the lung-fields lack contrast and assume a "veiled" appearance, which has been likened to that seen on looking through ground-glass; (4) in a lessened susceptibility to the supervention of pulmonary tuberculosis compared with silicosis (though these cases more frequently contract pneumonia as a complication); and (5) in the early appearance in the sputum, and in the lungs themselves, of the peculiar "asbestos bodies," which are yellowish-brown, of elongated bead-like form, often with bulbous ends.

### SUMMARY.

The typical results of the inhalation of finely-divided silica dust over long periods of time, or in strong concentrations, is first to produce what may be called a chronic lymphatic congestion, with possibly some bronchiolitis, a stage from which recovery may be expected with little or no disability or fibrosis; then to produce the simple nodular "dust-fibrosis" which is silicosis; and, finally, in most cases to lead on to a condition of "infective silicosis" and to a true "dust-phthisis."

Individual cases show many variations in character and mode of development, depending on the factors of time, intensity and infection, and on the nature of the dust inhaled.

Pathological changes in the lymph-nodes previously effected in early life by tubercle and other agencies, together with the initial physique and the degree of inherited or acquired resistance to infection, all have an influence on the rapidity of onset and progression of the disease.

In conclusion, I am indebted to Dr. Sutherland of the Home Office, and to Drs. Keating and McVittie of the Bristol Silicosis Board, for permission to show some of the skiagrams which I have made for the Board; also to Dr. Peter Kerley for the loan of one or two additional skiagrams, and to the South African Mine Medical Officers' Association for much valuable material, including the beautiful pathological plates which you have seen.

The fine radiographic details illustrating the pathological changes in the lungs, particularly in the early stages of the disease, have unfortunately been to a great extent lost in the process of reproduction of the original skiagrams.

### BIBLIOGRAPHY.

L. R. Sante, M.D., "The Chest," *Annals of Rœntgenology*, vol. xi.

"Silicosis in South Africa," *Proceedings of the Transvaal Mine Medical Officers' Association*, 1930.

J. S. Haldane, *Silicosis and Coal Mining.*

E. L. Middleton, M.D., "A Study of Pulmonary Silicosis," *Jour. Indust. Hyg.*, 1920–21, ii. 433–449.

Merewether and Price, *Report on Effect of Asbestos Dust in the Lungs*, H.M.S.O., 1930.

Sutherland, Bryson and Keating, *Report on the Occurrence of Silicosis amongst Granite Workers.*

Sutherland and Bryson, *Report on the Occurrence of Lung Disease from Dust Inhalation in the Slate Industry*, H.M.S.O., 1930.

*The Quarry Managers' Journal*, vol. xiii., No. 2, May, 1930.

Pancoast and Pendergrass, "A Review of Pneumoconiosis," *American Journal of Rœntgenology*, October, 1931, xxvi. 556.

Gye and Kettle, *Lancet*, 1922, ii. 855.

Gye and Purdy, *Brit. Jour. Exper. Path.*, 1922, iii. 75, iii. 86; 1924, v. 238.

## Figures and Tables

**Fig. 1. f1:**
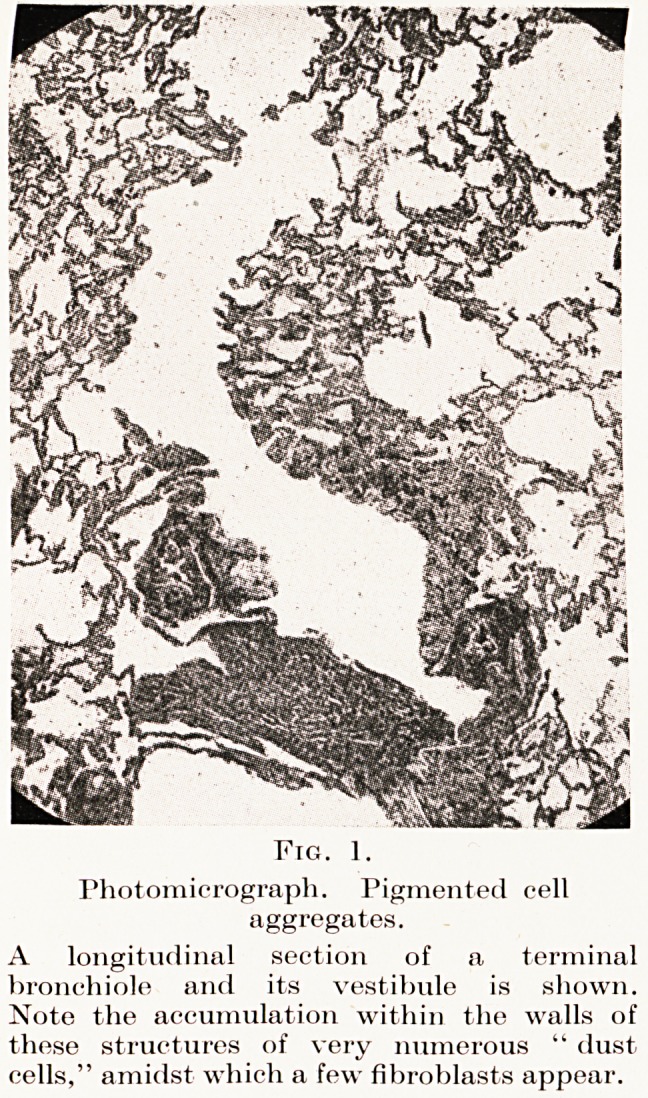


**Fig. 2. f2:**
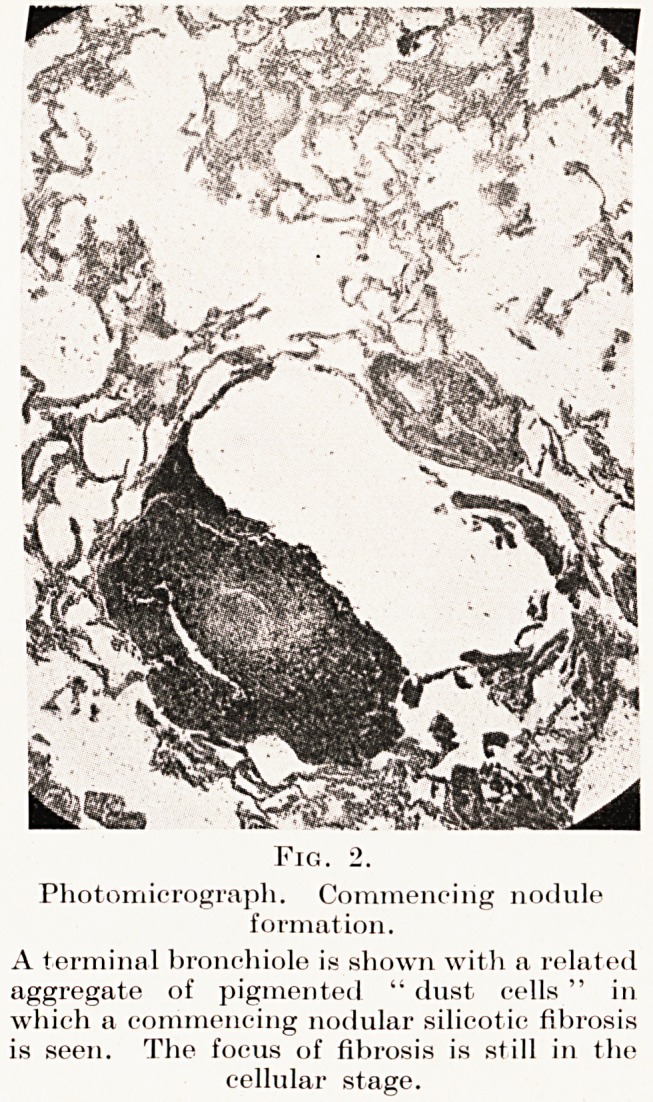


**Fig. 3. f3:**
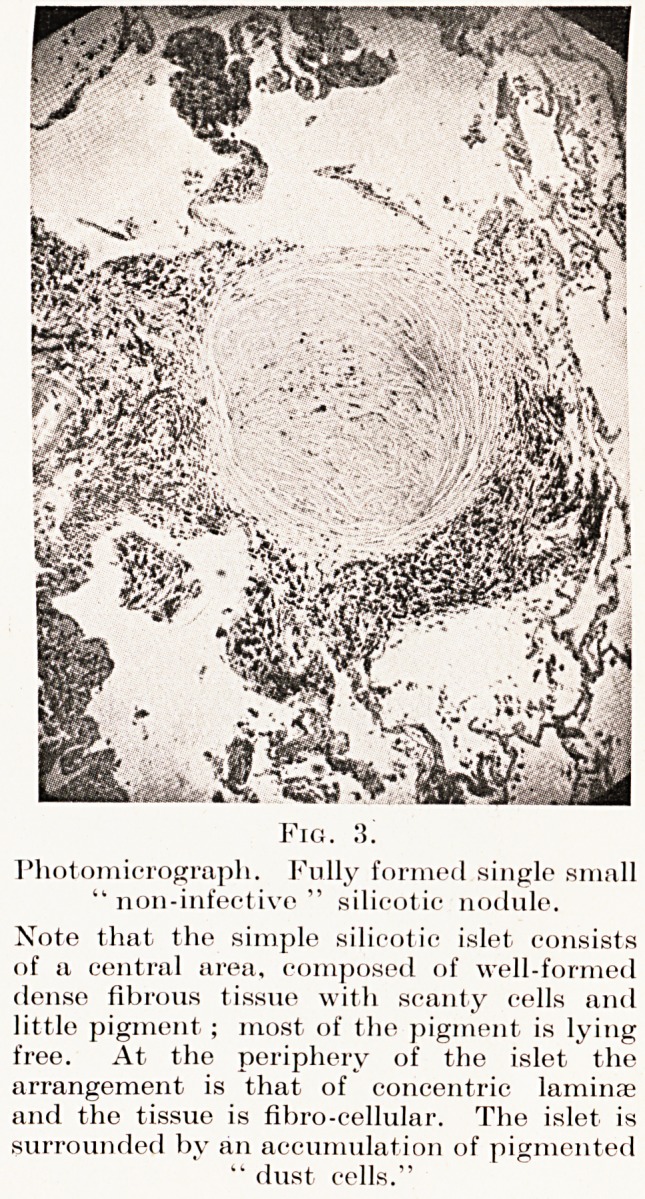


**Fig. 4. f4:**
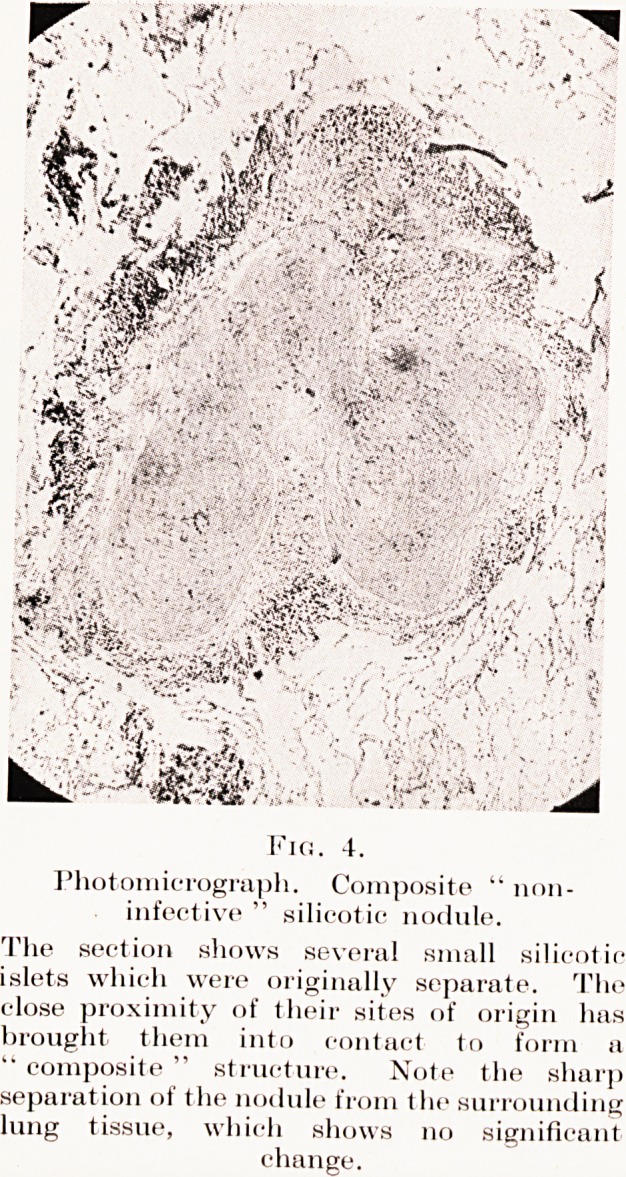


**Fig. 5. f5:**
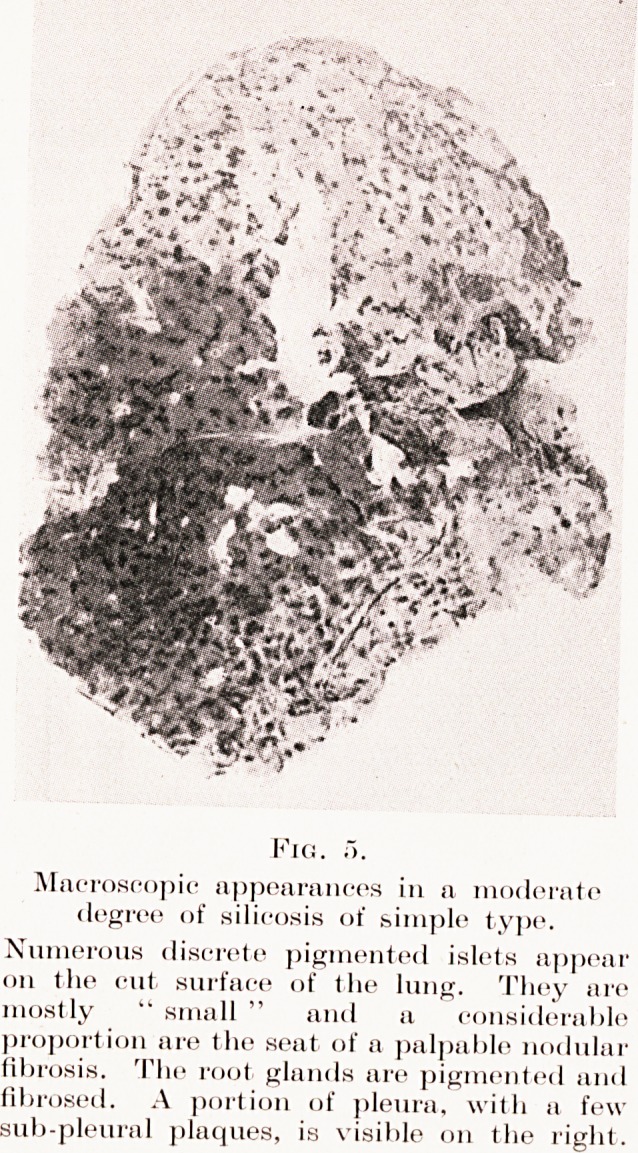


**Fig. 6. f6:**
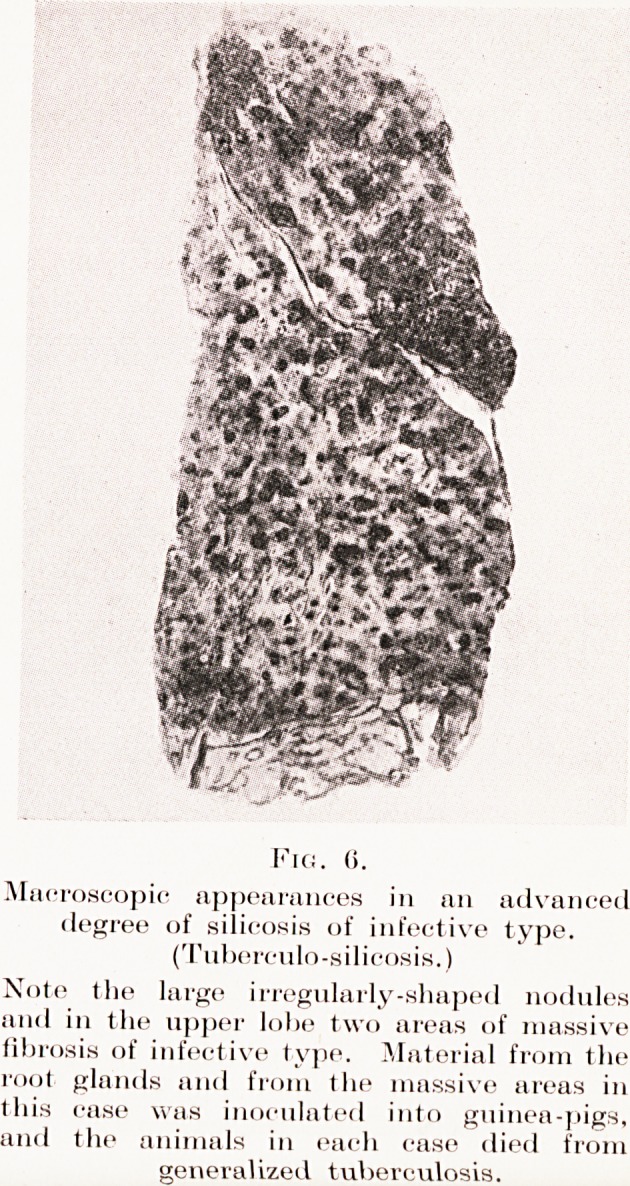


**Fig. 7. f7:**
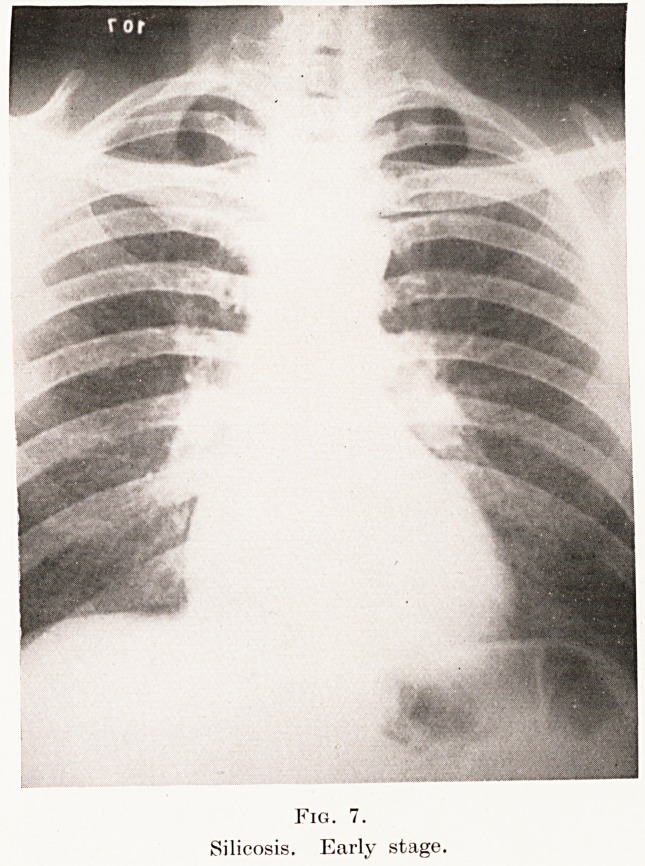


**Fig. 8. f8:**
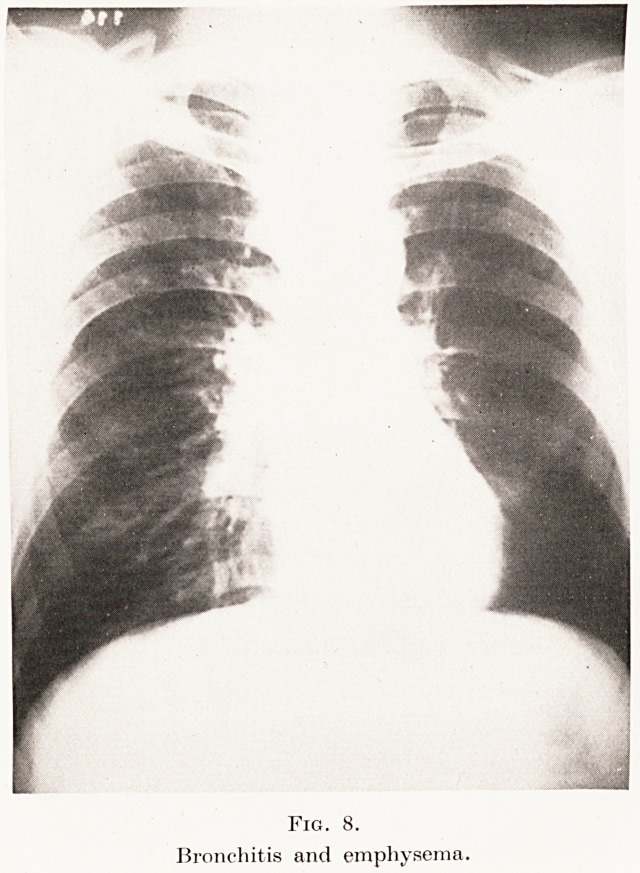


**Fig. 9. f9:**
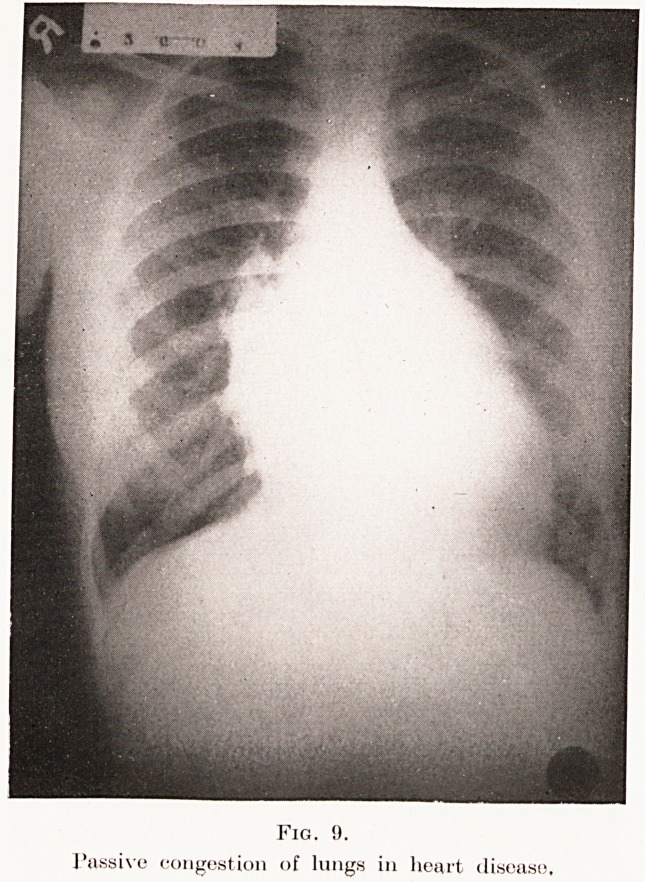


**Fig. 10. f10:**
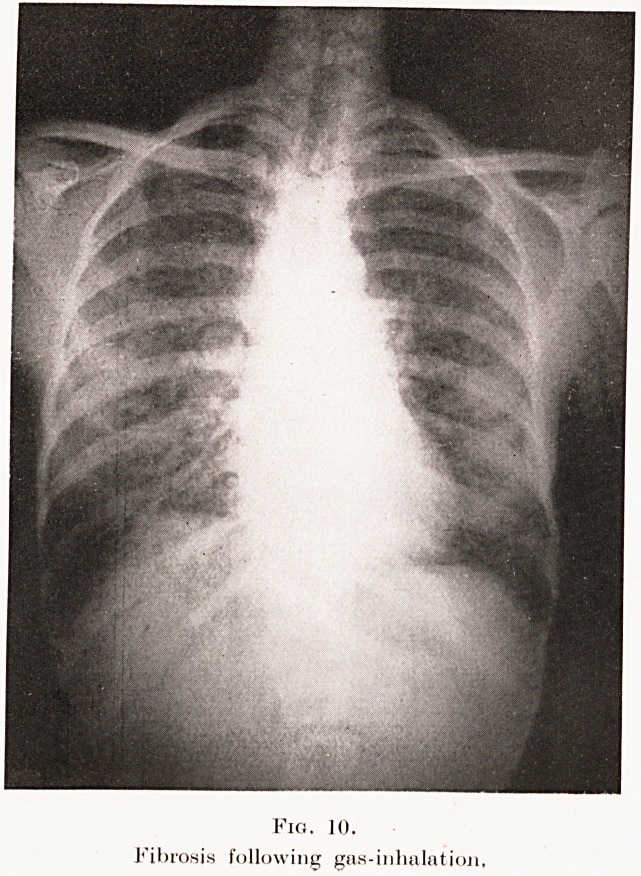


**Fig. 11. f11:**
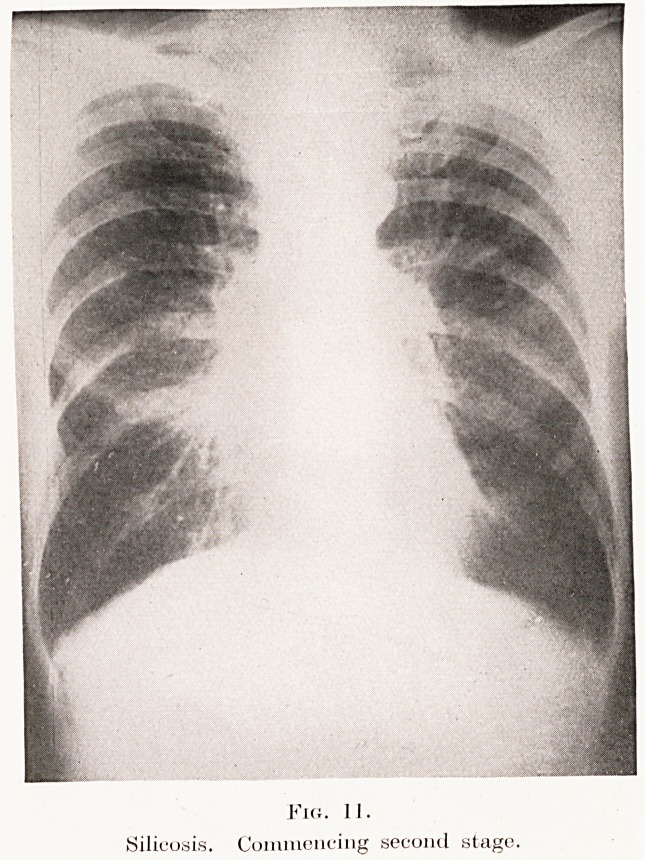


**Fig. 12. f12:**
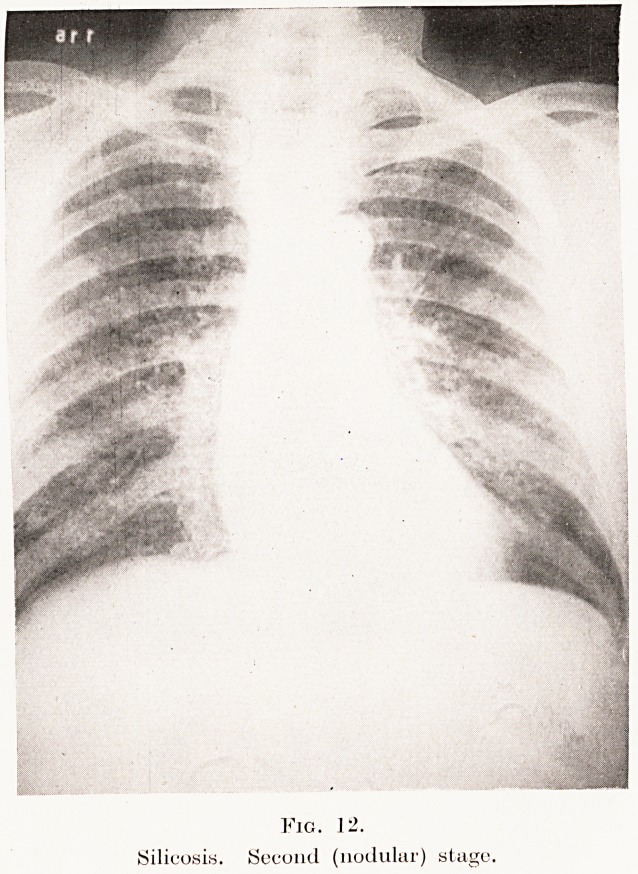


**Fig. 13. f13:**
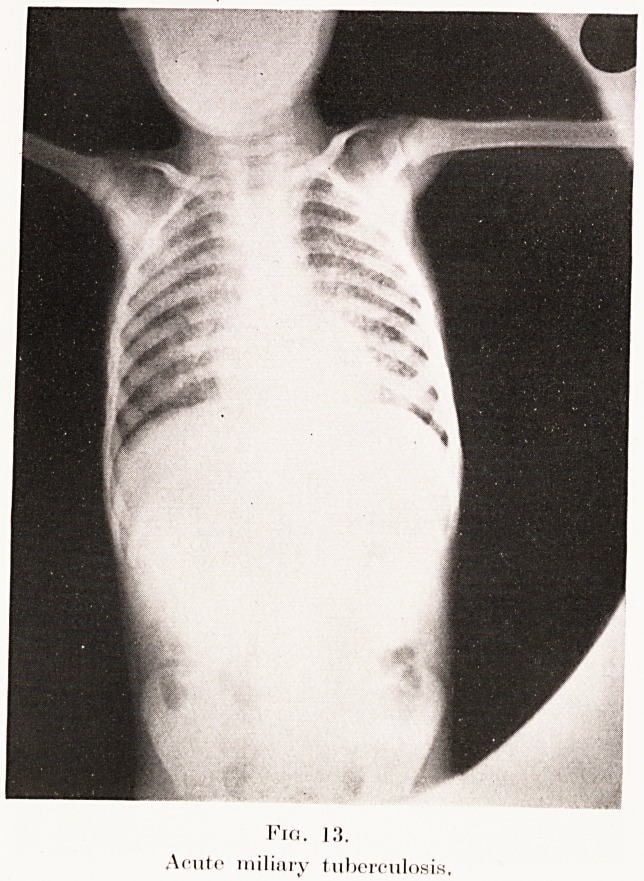


**Fig. 14. f14:**
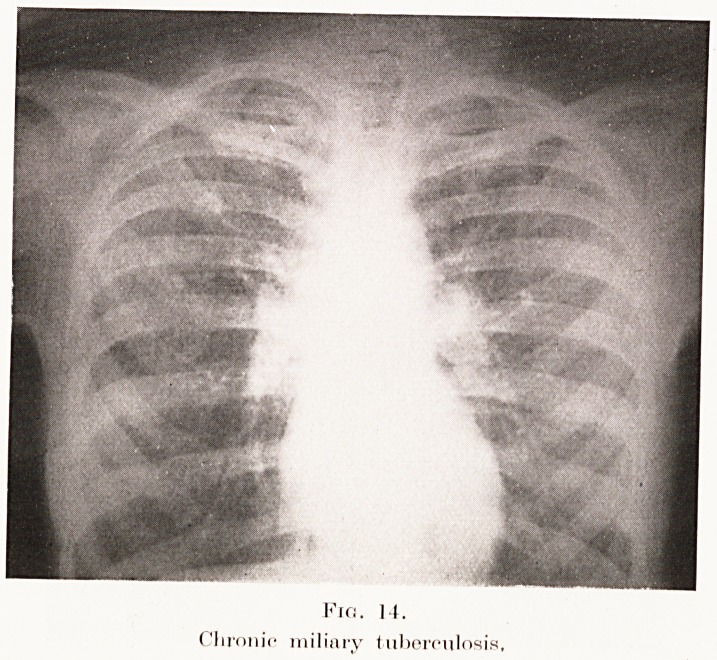


**Fig. 15. f15:**
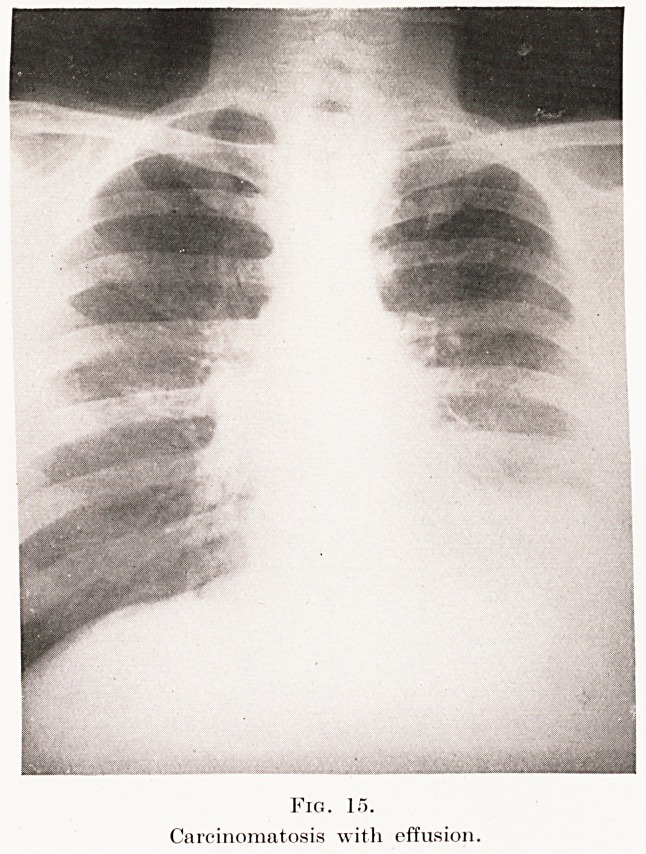


**Fig. 16. f16:**
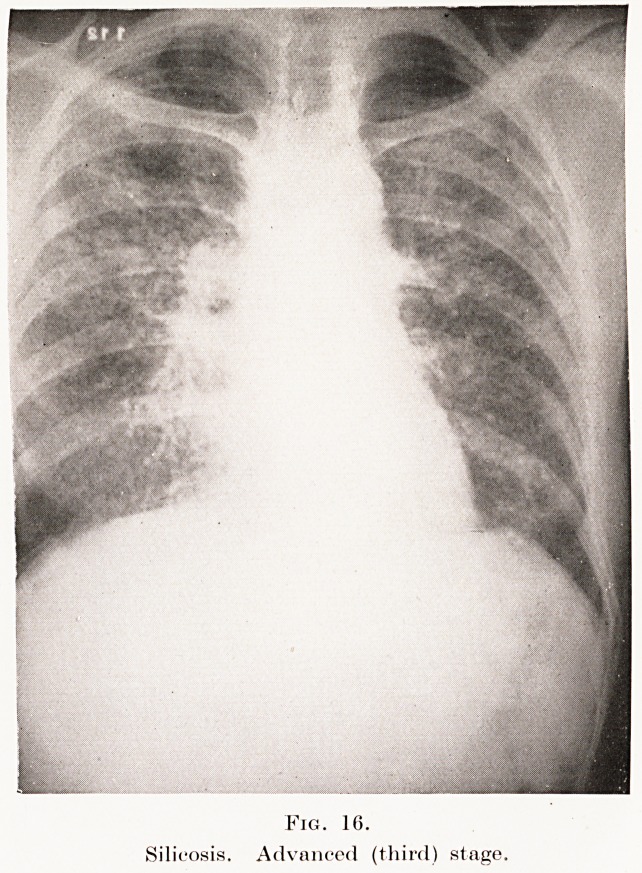


**Fig. 17. f17:**
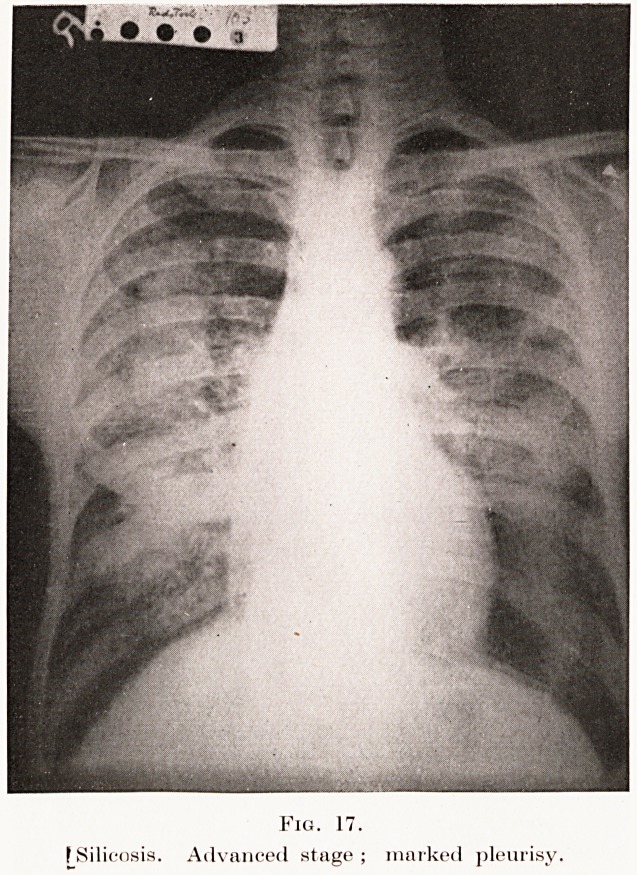


**Fig. 18. f18:**
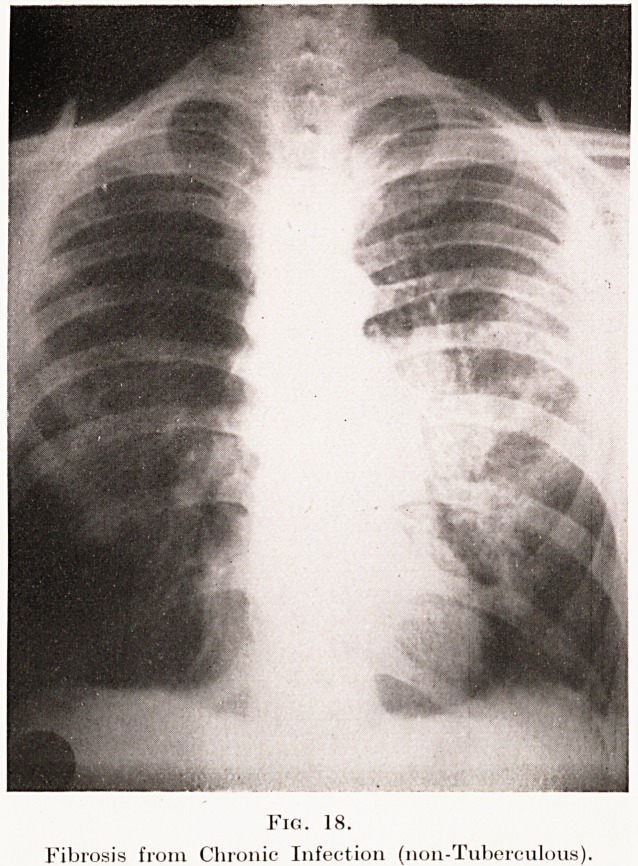


**Fig. 19. f19:**
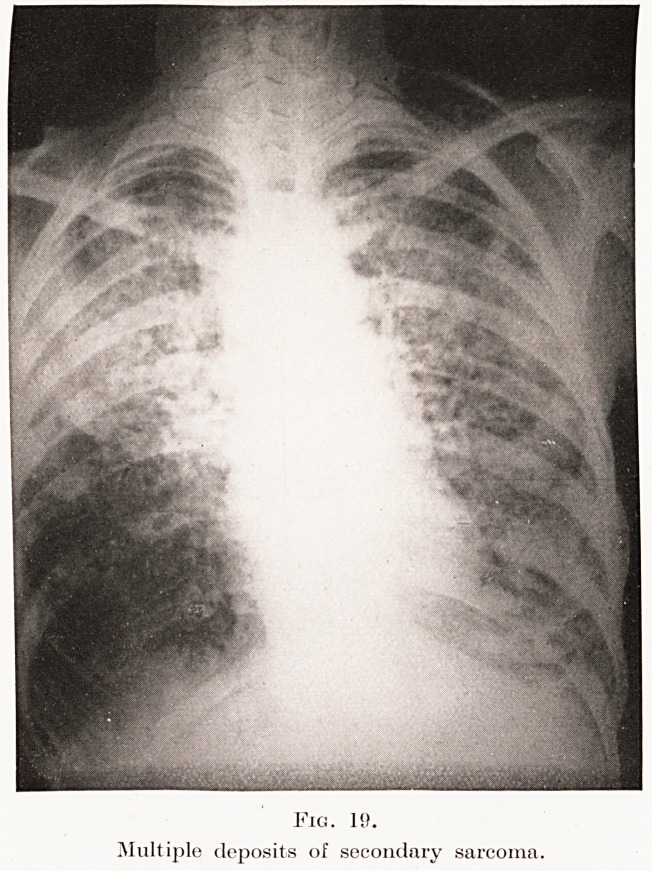


**Fig. 20. f20:**
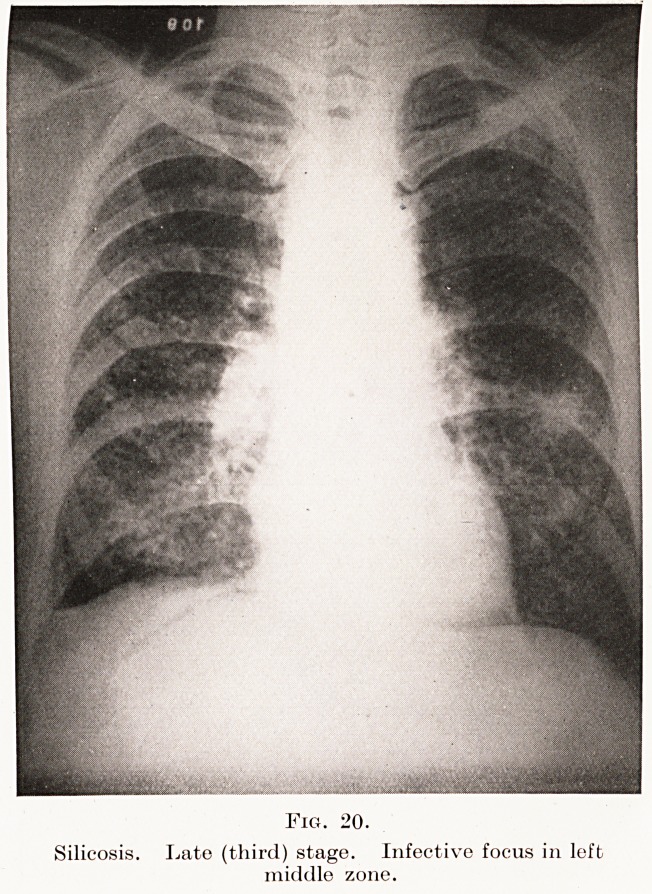


**Fig. 21. f21:**
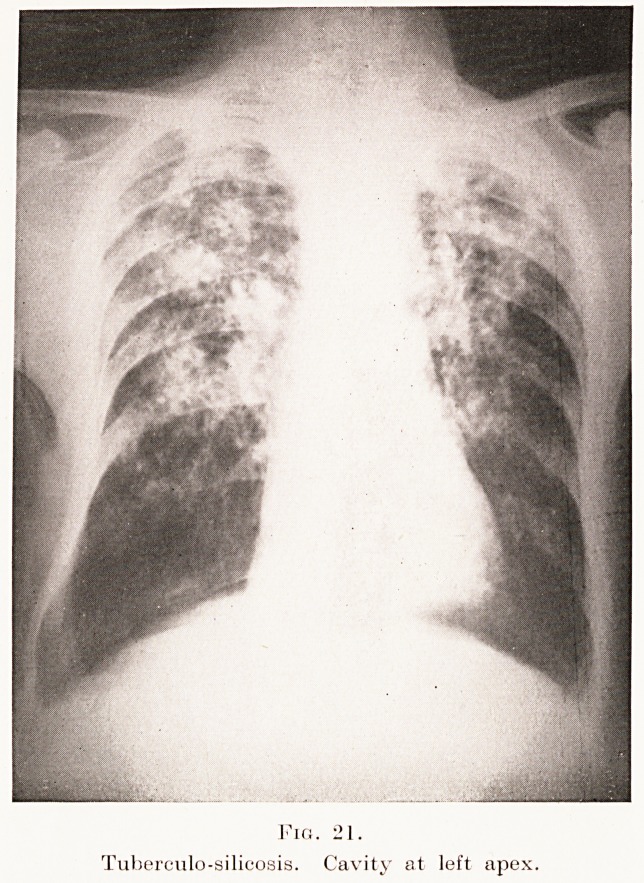


**Fig. 22. f22:**